# Complete Genome Sequence of Novel Psychrotolerant *Legionella* Strain TUM19329, Isolated from Antarctic Lake Sediment

**DOI:** 10.1128/MRA.00253-20

**Published:** 2020-04-16

**Authors:** Sho Shimada, Ryosuke Nakai, Kotaro Aoki, Norifumi Shimoeda, Giichiro Ohno, Yasunari Miyazaki, Sakae Kudoh, Satoshi Imura, Kentaro Watanabe, Yoshikazu Ishii, Kazuhiro Tateda

**Affiliations:** a Department of Microbiology and Infectious Diseases, Toho University School of Medicine, Tokyo, Japan; b Bioproduction Research Institute, National Institute of Advanced Industrial Science and Technology (AIST), Hokkaido, Japan; c Tochigi Medical Center, Tochinoki hospital, Tochigi, Japan; d Tokatsu Hospital, Chiba, Japan; e Department of Respiratory Medicine, Tokyo Medical and Dental University, Tokyo, Japan; f National Institute of Polar Research, Research Organization of Information and Systems, Tokyo, Japan; g Department of Polar Science, School of Multidisciplinary Sciences, SOKENDAI (The Graduate University for Advanced Studies), Tokyo, Japan; Georgia Institute of Technology

## Abstract

Here, we report the complete genome sequence characteristics of *Legionella* strain TUM19329, a candidate for a novel psychrotolerant species isolated from Antarctic lake sediment. The genome assembly contains a single 3,750,805-bp contig with a G+C content of 39.1% and is predicted to encode 3,538 proteins.

## ANNOUNCEMENT

*Legionella* spp. are ubiquitous Gram-negative intracellular bacteria that inhabit aquatic environments. The growth temperature of known *Legionella* strains is generally between 20°C and 43°C (1, 2), while culture-independent analysis has shown a large genetic diversity of uncultured *Legionella* spp. in water with a low temperature (∼15°C) (3) and their presence even in cold freshwater lakes (near 0°C) of King George Island in the Antarctic Peninsula (4). However, it is still unclear whether cold-adapted species, i.e., psychrophilic or psychrotolerant species, exist in low-temperature environments. In this context, we attempted to isolate *Legionella* spp. inhabiting Antarctic cold environments by using the enrichment method (5) with minor modifications. Briefly, 50 g of a sediment sample with 100 ml distilled water was incubated at 4°C instead of 33°C for 3 months. Subsequently, the incubated sample was subcultured on Wadowsky-Yee-Okuda agar plates (6) supplemented with α-ketoglutarate (Eiken Chemical Co., Ltd., Tokyo, Japan) for 2 weeks. Next, *Legionella*-like colonies were picked up and identified. We then obtained the first psychrotolerant strain (growth range, 4°C to 25°C), designated TUM19329, from the sediment of Lake Naga-ike in the Skarvsnes ice-free area in East Antarctica (0°C to 10°C; 69°29′S, 39°35′E) (7). Here, we report the complete genome sequence and the molecular characterization of this unique strain.

For genomic characterization, the isolate TUM19329 was grown on buffered charcoal-yeast extract α-ketoglutarate agar for 10 days at 25°C. Whole-genome sequencing was performed using MinION (Oxford Nanopore Technologies, Oxford, United Kingdom) and MiSeq (Illumina, San Diego, CA, USA) instruments. DNA extraction, library preparation, and MinION sequencing were performed with the NucleoBond AXG 20 column (TaKaRa Bio, Shiga, Japan) combined with the NucleoBond buffer set III (TaKaRa Bio), 1D rapid barcoding kit (catalog number SQK-RBK004; Oxford Nanopore Technologies), and R9.4 flow cell (Oxford Nanopore Technologies), respectively. Base calling was performed using Guppy (version 2.3.7). DNA extraction, library preparation, and Illumina MiSeq sequencing were performed using a QIAamp DNA minikit (Qiagen, Venlo, Netherland), a QIAseq FX DNA library kit (Qiagen), and a MiSeq reagent kit v3 with 300-bp paired-end reads (Illumina), respectively. Approximately 60,000 MinION reads and 400,000 MiSeq reads were obtained for the isolate. Hybrid *de novo* assembly was conducted using Unicycler (version 0.4.0) (8) after MinION and MiSeq raw reads were quality filtered and adapter trimmed with NanoFilt (version 2.5.0) (quality score cutoff of 6 and minimum length of 5,000 bp) (9) and the Trimmomatic tool (quality score cutoff of 20) (10), respectively. The contigs were polished three times by Pilon (11). Default parameters were used for all software, unless otherwise noted.

The final assembly resulted in one large closed chromosome with a total length of 3,750,805 bp. Average sequence depth was 345×. Annotation was performed using DFAST (version 1.1.0) with standard settings (12), which predicted a genome encoding 3,538 proteins and a G+C content of 39.1%. To assess similarity, 16S rRNA gene sequences were searched using blast against the NCBI sequence database, and average nucleotide identity (ANI) was calculated with FastANI (13) against genomes of *the* genus *Legionella* downloaded from the NCBI RefSeq database in March 2020. Strain TUM19329 showed relatively low 16S rRNA gene sequence similarity (<97.5%) and ANI (<78%) values for all known *Legionella* species. Considering the threshold for taxonomic assignment (14), our data suggest that this strain is a candidate novel species of the genus *Legionella*. However, the strain shared high 16S rRNA gene sequence similarity (99.9%) with the environmental sequence (DDBJ/ENA/GenBank accession number AB630760) recovered from a benthic moss colony (moss pillar) of another freshwater lake in East Antarctica (15). These results suggest the possibility that cold-adapted *Legionella* members are present in other cold freshwater sources.

### Data availability.

The genome sequence of *Legionella strain* TUM19329 was deposited in DDBJ/ENA/GenBank under the accession number AP022839. Raw sequence data for this strain were deposited under SRA accession numbers DRR213975 (MiSeq) and DRR213976 (MinION).
